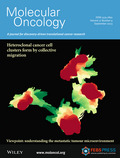# Issue Information

**DOI:** 10.1002/1878-0261.13239

**Published:** 2023-09-07

## Abstract

On the cover: Distant cancer cell aggregates exhibit directional collective migration by emitting actin‐rich protrusions that extend towards neighbouring aggregates, thus forming multiclonal tumors. Read the full article by Miriam Palmiero *et al.* in pp. 1699–1725.

The content included in the second part of this issue, was selected to support the “6th meeting on Advances in Circulating Tumor Cells” related to the call for content on **Liquid Biopsy and Precision Oncology**. Read the full articles starting from pp. 1844–1856.